# A modelling approach to disentangle the factors limiting muscle oxygenation in smokers

**DOI:** 10.1007/s00421-023-05289-y

**Published:** 2023-08-06

**Authors:** Hans Degens, Tomas Venckunas, Rob Cl Wüst

**Affiliations:** 1 Department of Life Sciences, Research Centre for Musculoskeletal Science and Sports Medicine, Manchester Metropolitan University, John Dalton Building, Chester Street, Manchester, M1 5GD UK; 2 Lithuanian Sports University, Kaunas, Lithuania; 3 Laboratory of Myology, Department of Human Movement Sciences, Faculty of Behavioural and Movement Sciences, Amsterdam Movement Sciences, Vrije Universiteit Amsterdam, Amsterdam, The Netherlands

**Keywords:** Smoking, Skeletal muscle, Capillarisation, Oxygenation

## Abstract

Cigarette smoking is associated with a lower exercise capacity and lower muscle fatigue resistance. This is at least partly attributable to carboxyhaemoglobin (HbCO) in the blood that via reduction in the oxygen-carrying capacity, and the left-shift of the Hb-dissociation curve would reduce tissue oxygenation. On the other hand, a reduced oxygen uptake due to mitochondrial dysfunction would result in improved oxygenation. We used previously collected capillarisation, myoglobin and estimated cellular maximal muscle oxygen consumption data derived from succinate dehydrogenase-stained sections from the vastus lateralis muscle from six smokers and five non-smokers. These data were fed into an expanded Krogh tissue oxygenation model to assess whether an impaired muscle fatigue resistance in smokers is primarily due to HbCO or impaired mitochondrial respiration. The model showed that in smokers with 6% and 20% HbCO (causing a left-shift of the Hb-dissociation curve) average muscle oxygenation was reduced by 1.9% and 7.2%, respectively. Muscle oxygenation was increased by 13.3% when maximal mitochondrial respiration was reduced by 29%. A combination of a 29% reduction in maximal mitochondrial respiration and 20% HbCO led to no significant difference in muscle oxygenation from that in non-smokers. This indicates that while HbCO may explain the reduced exercise capacity after just one smoking session, in chronic smokers impaired mitochondrial respiration appears more important in reducing oxygen extraction and exercise capacity with only a small contribution of the left-shift of the Hb-dissociation curve.

## Introduction

Cigarette smoking is an important risk factor for chronic obstructive pulmonary (COPD) (Eisner et al. 2010) and cardiovascular (Morris et al. 2015) diseases. Yet, many continue to smoke, reflecting addiction to cigarette smoking, attributable to—among others—benefits to attention and memory, and nicotine withdrawal effects hampering smoking cessation (Heishman et al. 2010). While nicotine may enhance exercise capacity (Mundel and Jones 2006), it has been seen that smoking, in otherwise non-smokers, acutely reduces the exercise capacity (Hirsch et al. 1985). This reduced exercise capacity was associated with a reduced oxygen uptake per heart beat (‘oxygen pulse’) during sub-maximal and maximal exercise (Hirsch et al. 1985), suggesting an impaired peripheral oxygen extraction during exercise.

An acutely impaired oxygen extraction may result from the combination of carbon monoxide (CO) with haemoglobin (Hb) that will not only reduce the oxygen-carrying capacity of the blood but also, via a left-shift of the haemoglobin-dissociation curve, impair the release of oxygen to the working muscle (Degens et al. 2015). That such a mechanism may play a role is reflected by the lower muscle fatigue resistance during a series of electrically evoked contractions in smokers (Wust et al. 2008b) that was also seen after exposure to CO resulting in 6% HbCO (Morse et al. 2008), a level typically seen in smokers (McDonough and Moffatt 1999). Like its effects on haemoglobin, CO may also reduce the oxygen-carrying capacity of myoglobin (Mb), impairing the Mb-facilitated diffusion and intracellular buffering of oxygen, and consequently diminishing the delivery of oxygen to mitochondria (Wittenberg 1970). These impacts on oxygen delivery may significantly reduce muscle oxygenation during contractile activity and hamper aerobic ATP generation, important for fatigue resistance (Degens and Veerkamp 1994), particularly, if the pO_2_ that mitochondria experience falls below 7.5 mmHg (1 kPa) where it will start to impair mitochondrial oxygen consumption (Donnelly et al. 2022).

Besides these acute effects of CO in cigarette smoke, components in cigarette smoke may also contribute to a diminished mitochondrial respiration, often seen in smokers (Ajime et al. 2021; Alonso et al. 2004; Cardellach et al. 2003; Larsson and Orlander 1984). CO can bind to cytochrome oxidases, directly inhibiting mitochondrial respiration at complex IV (Alonso et al. 2003), and acutely reduce skeletal muscle mitochondrial respiration in a dose-dependent manner (Ajime et al. 2021). A diminished mitochondrial respiration may, like the formation of HbCO, underlie the impaired oxygen extraction seen in smokers; impaired mitochondrial respiration via reduced use, and HbCO via reduced delivery, of oxygen to the working muscle. In other words, the CO-induced left-shift and associated reduced oxygen-carrying capacity will result in a diminished muscle oxygenation, while mitochondrial dysfunction may improve oxygenation, while both will impair oxygen extraction. Therefore, the direction of smoking-induced changes in muscle oxygenation during maximal exercise may help identify the relative significance of mitochondrial dysfunction and impaired oxygen delivery for the diminished oxygen extraction, muscle fatigue resistance and exercise capacity in smokers.

The aim of the present study was, therefore, to use a muscle oxygenation model (Wust et al. 2009) to assess the potential impact of CO via reducing the oxygen-carrying capacity of Hb and Mb and the left-shift of the Hb-dissociation curve with or without mitochondrial dysfunction, combined with previously observed muscle capillarisation, and maximal cellular oxygen consumption and myoglobin concentration of individual fibres in smokers and non-smokers (Wust et al. 2008a), on muscle oxygenation. This will help to unravel the relative importance of limitations in oxygen delivery and cellular respiration for the diminished muscle fatigue resistance and exercise capacity in smokers.

## Materials and methods

This study used histological data from muscle biopsies of 5 controls and 6 smokers (Table 1) that were published previously (Wust et al. 2008a). The study was approved by the local Manchester Metropolitan University ethics committee (Ref no: 2005/02/03) and each participant provided written informed consent before the biopsy was taken.

**Table 1:** Participant characteristics and muscle morphological data

	Non-smokers (2 men; 3 women)	Smokers (3 men; 3 women)
Age (y)	55.2 ± 12.3 (43–73)	41.6 ± 22.7 (20–72)
Height (m)	1.76 ± 0.09	1.71 ± 0.06
Body mass (kg)	76.5 ± 14.4	70.0 ± 6.0
BMI (kg m^−2^)	24.4 ± 3.2	24.4 ± 1.4
Cigarettes/week	0	108 ± 65
CD (mm^−2^)	345 ± 107	339 ± 138
Log_R_SD	0.100 ± 0.015	0.104 ± 0.038
VO_2_max (nmol mm^−3^ s^−1^)	0.082 ± 0.018	0.076 ± 0.019
[Mb] (mM)	0.645 ± 0.042	0.656 ± 0.050

Data are mean ± SD

*BMI* body mass index; *CD* capillary density; *Log*_*R*_*SD* logarithmic standard deviation of the oxygen supply areas (defined as the area around a capillary delineated by equidistant boundaries from adjacent capillaries; an indicator of the heterogeneity of capillary spacing (Degens et al. 2006a)); *VO*_*2*_*max and [Mb]* average maximal oxygen consumption and myoglobin concentration of the fibres in a given muscle section, determined by quantitative SDH and myoglobin histochemistry, respectively

To study the effects of different levels of carboxy haemoglobin (COHb; 0, 6 and 20%, typically seen in smokers (Dorey et al. 2020)) on muscle tissue oxygenation during maximal muscle oxygen consumption, we applied an expansion of the Krogh model (Oxytis, the Netherlands) (Hoofd 1995a, b) on data obtained from histological cross sections of vastus lateralis muscle biopsies from smokers and non-smokers. The mathematical aspects of the study have been published before (Hoofd 1995a, b), and can be obtained from https://hoofd.info/louis/s/s_Apps.html. While smoking is accompanied with vascular dysfunction (Celermajer et al. 1993) that will result in a lower blood flow and, hence, oxygen delivery to the working muscles, we assumed in our model that blood flow was not altered by smoking. This allowed us to calculate the distribution of tissue pO_2_ dependent on maximal oxygen consumption of the muscle tissue and/or HbCO levels independent of smoking-induced changes in muscle blood flow. To calculate the oxygen tension distribution in the tissue, we fed into the model the capillarisation, average Mb concentration and a homogeneous maximal oxygen consumption of individual muscle fibres, determined with quantitative succinate dehydrogenase (SDH) histochemistry presented in another publication on the same samples (Wust et al. 2008a), and using the correlation between SDH activity and maximal fibre oxygen consumption (Van der Laarse et al. 1989, 2005**)**. The PO_2_ at the start of the capillary was set at 95 mmHg and the tissue PO_2_ was assessed in a tissue slab with a thickness of 400 µm that was divided in 40 layers with a 10- µm grid distance. The oxygen permeability, also called the Krogh’s diffusion coefficient, for muscle tissue was set at 6 × 10^–12^ mol·m^−1^ mmHg^−1^·s^−1^ (van der Laarse et al. 2005). The Mb-facilitated pressure was calculated using the measured myoglobin concentration (Wust et al. 2008a) and the diffusion coefficient for myoglobin in muscle tissue (0.27 × 10^–10^ m^2^·s^−1^) (Des Tombe et al. 2002). As previously (Degens et al. 2006a), the transfer factor (average capillary blood flow (µm^3^ s^−1^) divided by the tissue's oxygen diffusion coefficient (µm^2^ s^−1^)) and extraction pressure were 75 µm and 5 mmHg, respectively. The maximal oxygen consumption of the muscle fibres was determined from the average absorbance of the SDH staining (A_660_) (Des Tombe et al. 2002):

$${\text{VO}}_{{2}} {\text{max }}({\text{nmol mm}}^{{ - {3}}} {\text{ s}}^{{ - {1}}} ) \, = { 6}000 \, \times {\text{ A}}_{{{66}0}} /\left( {{\text{section thickness }}\left( {{\mu m}} \right) \, \times {\text{ incubation time }}\left( {\text{s}} \right)} \right).$$

As the %MbCO parallels the %HbCO (McDonough and Moffatt 1999), the maximal Mb oxygen saturation was set at 100%, 94% and 80% for 0%, 6% and 20% HbCO, respectively. In addition, the oxygen capacity pressure (Pcap) and Mb facilitation pressure were reduced in proportion to the Hb and Mb oxygen-binding sites occupied by CO, taking into account for Pcap that approximately 3 mL O_2_ is dissolved per L arterial blood at 95 mmHg. The binding of CO to Hb does not only reduce the oxygen-carrying capacity, but also results in a left-shift in the Hb-dissociation curve, reflected by a decrease in PO_2_ at which the Hb saturation is 50% (P50) and a decrease in the Hill coefficient ‘*n*’. The P50 at a given %COHb was derived from published data (Hlastala et al. 1976) and the ‘*n*’ for a given %COHb (n*) was calculated as:

$$n^{*} = n\left( {{1} + F_{{{\text{CO}}}} } \right)/\left( {{1} + nF_{{{\text{CO}}}} } \right),$$

where F_CO_ is the fraction of Hb occupied by CO. The values used for the different proportions of COHb are given in Table 2. In addition, we calculated the impact of a reduced maximal oxygen consumption of the mitochondria on tissue oxygenation, assuming a 29% reduction in maximal complex II respiration (SDH), as seen in muscle tissue from mice exposed to long-term cigarette smoking (Ajime et al. 2021).

**Table 2:** The Hill coefficient (*n*), oxygen capacity pressure (Pcap) and oxygen pressure at which haemoglobin (P50Hb) is saturated for 50% at different proportions of carboxyhaemoglobin (COHb) used to model muscle oxygenation

%COHb	*n*	Pcap (mmHg)	P50Hb (mmHg)
0	2.72	6667	27.2
6	2.48	6273	24.6
20	2.11	5353	19.7

### Statistics

Differences in average tissue oxygenation between smokers with 6% or 20% HbCO with or without mitochondrial dysfunction and non-smokers with 0% HbCO and normal mitochondrial function were analysed by ANOVA and a Dunnet post hoc test. If data were not normally distributed, a Kruskal–Wallis test was used. Differences were considered significant at *P* < 0.05. Data are presented as mean ± SD.

## Results

The participant characteristics and muscle morphology data are presented in Table 1. There were no significant differences in age, body mass, height or body mass index between smokers and non-smokers. Also, the capillary density, heterogeneity of capillary spacing (reflected by Log_R_SD; see legend for explanation), maximal oxygen consumption and myoglobin concentration of the skeletal muscle fibres did not differ significantly between smokers and non-smokers. Importantly, smokers had a lower fibre cross-sectional area (Wust et al. 2008a).

**Fig. 1:**
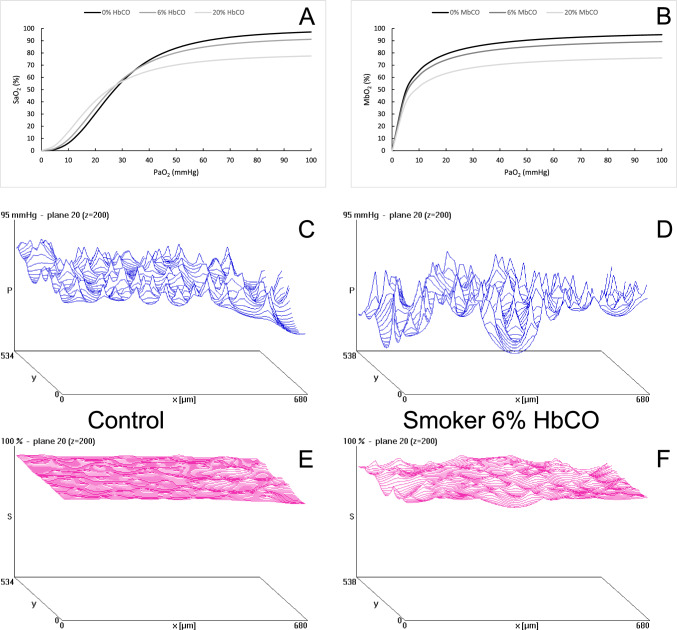
The impact of carbon monoxide on the (**A**) haemoglobin and (**B**) myoglobin dissociation curves. Oxygen tension profile 200 µm into a muscle tissue block working at maximal oxygen consumption in a non-smoker (**C**) and a smoker with 6% carboxyhaemoglobin (HbCO) (**D**). Panels **E** and **F** show the myoglobin saturation in the same tissue working at maximal oxygen consumption in a control and smoker, respectively

**Fig. 2:**
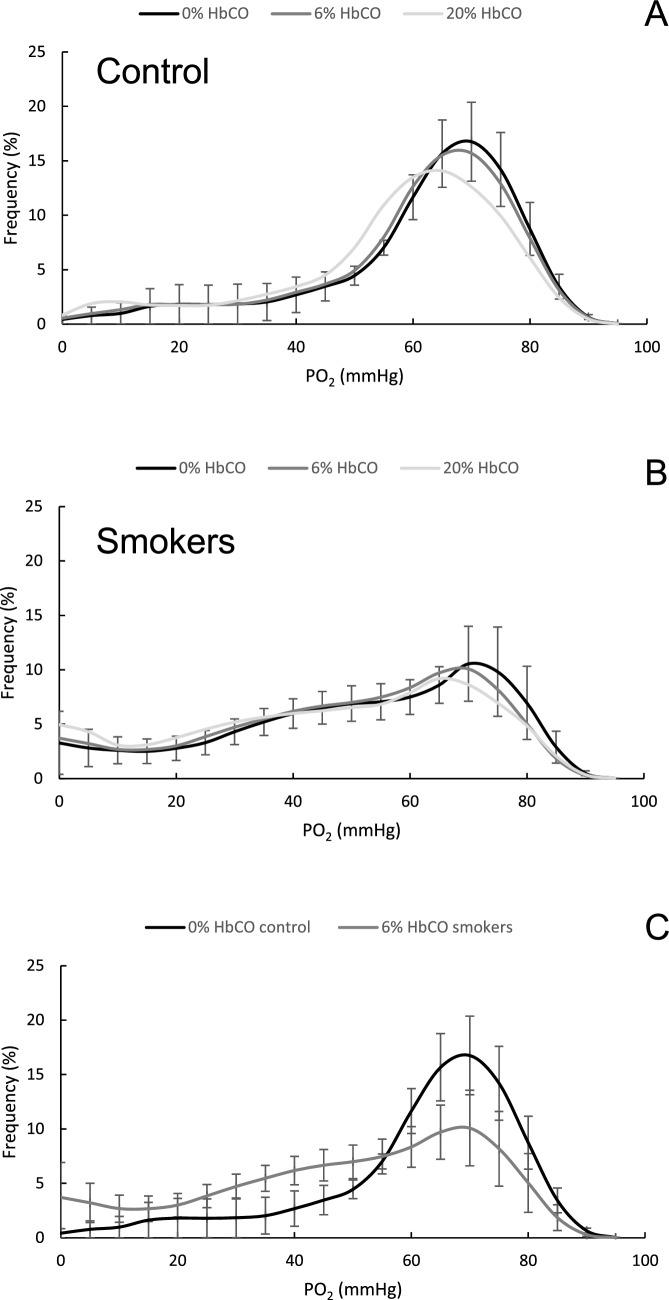
The impact of different levels of carboxyhaemoglobin (HbCO) and limitation of mitochondrial function (dotted lines) on the oxygen tension distribution in muscle from (**A)** non-smokers and (**B)** smokers working at maximal oxygen consumption. In panel **C,** the oxygen tension profile in muscles from non-smokers is compared with that of smokers with (i) 6% HbCO and (ii) 6%HbCO+ impaired mitochondrial function working at maximal oxygen consumption. Black: 0% HbCO; dark grey: 6% HbCO; light grey: 20% HbCO. Data are mean ± SEM

Figure 1A and B illustrates the reduced maximal oxygen saturation with increased HbCO for haemoglobin (Fig. 1A) and myoglobin (Fig. 1B), and the left-shift of the haemoglobin-dissociation curve (Fig. 1A). Figure 1C and D shows typical oxygen tension (PO_2_) profiles in muscle tissue, 200 µm downstream from arterial blood entering the tissue working at maximal oxygen consumption in a non-smoker (Fig. 1C) and smoker (Fig. 1D), respectively. Figure 1E and F shows the corresponding myoglobin saturation. In both controls (Fig. 2A) and smokers (Fig. 2B), a 29% reduction in the maximal oxygen uptake of the mitochondria improves tissue PO_2_, while an increase in the HbCO levels decreases tissue PO_2_. A reduced mitochondrial oxygen uptake more than compensates for the reduced tissue PO_2_ as a consequence of up to 20% HbCO. Figure 2C shows that there was no significant difference between the PO_2_ profile in muscles from non-smokers with normal mitochondrial function and 0% HbCO and that of smokers with 6% HbCO with or without mitochondrial dysfunction working at maximal oxygen consumption. Nevertheless, modelling showed that in controls, the average tissue PO_2_ was reduced by 1.9 and 7.2% by 6 and 20% HbCO, respectively, while it was elevated by 13.3% when maximal mitochondrial respiration was reduced by 29%

**Fig. 3:**
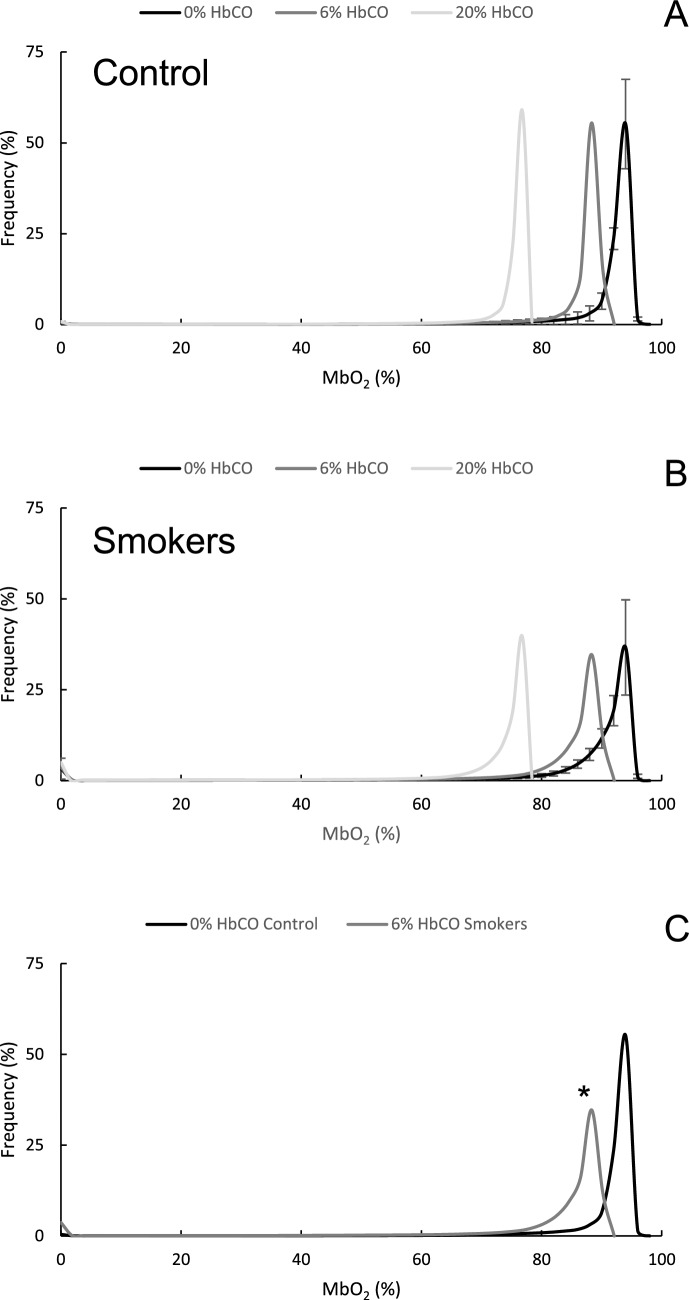
The impact of different levels of carboxyhaemoglobin (HbCO) and limitation of mitochondrial function (dotted lines) on the myoglobin saturation in muscle from (**A)** non-smokers and (**B)** smokers working at maximal oxygen consumption. In panel **C,** the myoglobin saturation in muscles from non-smokers is compared with that of smokers with (i) 6% HbCO and (ii) 6%HbCO+ impaired mitochondrial function working at maximal oxygen consumption. Black: 0% HbCO; dark grey: 6% HbCO; light grey: 20% HbCO Data are mean ± SEM. *: different from Control (C0) at *P* < 0.05

Figure 3 shows the impact of different levels of HbCO on the myoglobin saturation in muscle from non-smokers (Fig. 3A) and smokers (Fig. 3B) working at maximal oxygen consumption. Figure 3C shows that the myoglobin saturation in muscles from non-smokers was higher than that of smokers with 6% HbCO working at maximal oxygen consumption (*P* < 0.05). In controls, 6% HbCO reduced the myoglobin saturation by 5.8% points.

**Fig. 4:**
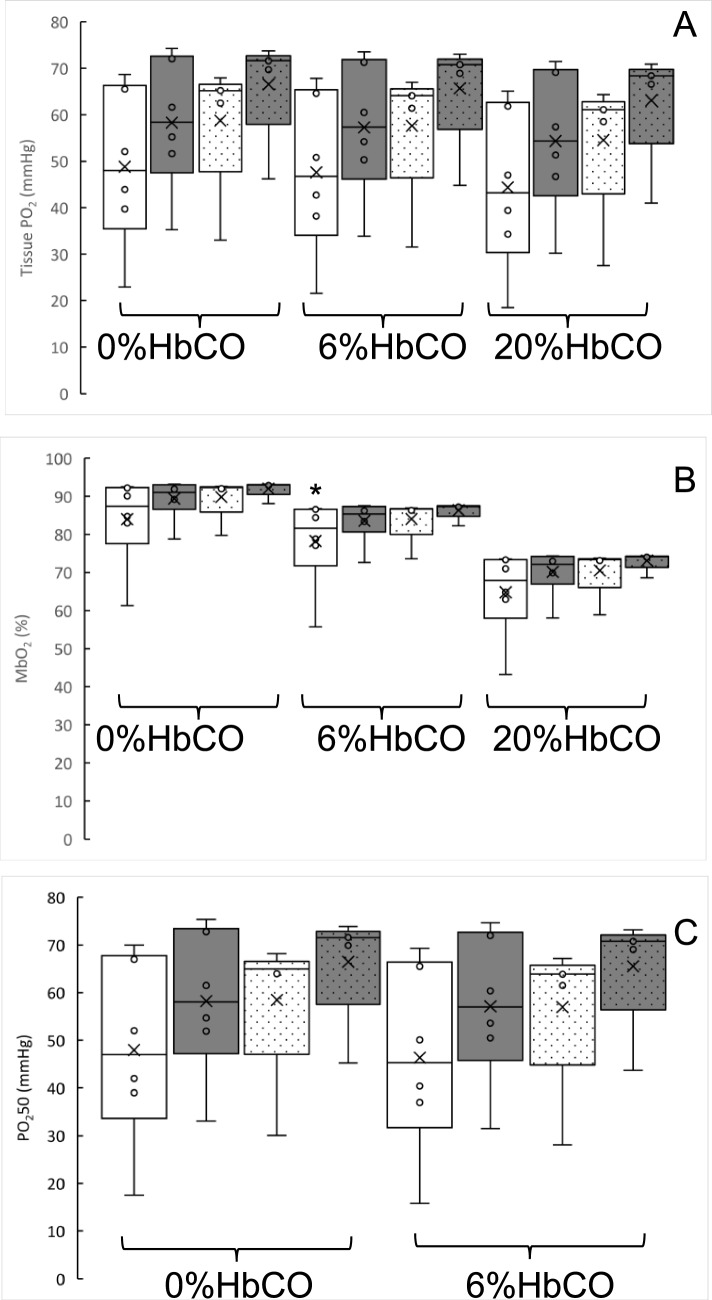
The impact of different levels (0, 6, 20%) of carboxyhaemoglobin (HbCO) and normal (white bars) and limited (grey bars) mitochondrial function in smokers and controls (dotted bars) on (**A**) the distribution of tissue PO_2_, (**B**) myoglobin saturation and (**C**) PO_2_50 in muscles from smokers (S) and non-smokers (C) working at maximal oxygen consumption. *: different from Control (C0) at *P* < 0.05

Figure 4 shows the impact of different levels (0, 6, 20%) of HbCO with or without impaired mitochondrial respiration on the distribution of tissue PO_2_ (Fig. 4A), myoglobin saturation (Fig. 4B) and P50 where 50% of the tissue has this or a lower PO_2_ in muscles from smokers. Impaired mitochondrial respiration resulted in a 13.3% higher tissue oxygenation, but there was no significant difference in the average tissue PO_2_ (Fig. 4A) or P50 (Fig. 4C) of smokers with 6% or 20% HbCO with or without impaired mitochondrial respiration from non-smokers (0% HbCO and normal mitochondrial respiration). The myoglobin saturation was less in smokers (assuming they had 6% or 20% HbCO) than non-smokers (*P* < 0.05; Figs. 3C, 4B).

## Discussion

The main observation of the present study was that muscle oxygenation in smokers with 6% and 20% circulating HbCO with or without mitochondrial dysfunction is not significantly lower than that in maximally working muscles from non-smokers with 0% HbCO and normal cellular respiration. However, modelling showed that if HbCO was increased to 6 or 20% the average PO_2_ was reduced by 1.9 and 7.2%, respectively, while a reduction of 29% in maximal cellular respiration resulted in a 13.3% rise in tissue oxygenation. This indicates that while HbCO may explain the reduced exercise capacity after just one smoking session, in chronic smokers impaired mitochondrial respiration appears more important in reducing oxygen extraction and exercise capacity with only a small contribution of the left-shift of the Hb-dissociation curve.

The absence of a significant difference in tissue oxygenation between smokers and non-smokers corresponds with our previous observation, using the Hill equation, of a non-significant difference in the critical PO_2_ (the interstitial PO_2_ that prevents an anoxic core in maximally working fibres) (Wust et al. 2008a) between smokers and non-smokers. While this is at least partly explicable by the smaller fibres in smokers (Wust et al. 2008a), neither the impact of a left-shift of the Hb-dissociation curve, nor the diminished Hb oxygen-carrying capacity due to formation of HbCO was considered. Surprisingly, even though 6 and 20% HbCO resulted in a 1.9 and 7.2% lower tissue oxygenation, respectively, including these levels of HbCO in the model still did not result in a significantly lower tissue oxygenation in smokers than non-smokers (0% HbCO), perhaps due to the large variation in tissue morphology (see Table 2). Nevertheless, it explains the observation that HbCO levels of up to 20% may not reduce the performance of sub-maximal exercise as oxygen delivery is compensated by an increase in cardiac output (Kane et al. 2016; Kizakevich et al. 2000).

While a decrease in tissue oxygenation by 1.9% may appear trivial, it has been seen that if maximal exercise results in haemoglobin desaturation by as little as 5% (6% HbCO already exceeds this level), it impairs maximal exercise capacity and oxygen consumption (Nielsen 2003). This effect in smokers is not only due to the lower oxygen-carrying capacity of the blood but also by the slower oxygen-dissociation kinetics as a consequence of the left-shift of the haemoglobin-dissociation curve (Rietbrock et al. 1992). In support of the importance of the left-shift of the haemoglobin-dissociation curve for maximal efforts is the observation that a reduction of the haemoglobin saturation to 80% by inhaling normobaric hypoxic air did not elicit a significant reduction in fatigue resistance during an identical series of electrically evoked contractions (Degens et al. 2006b), while just 6% HbCO—a much lower level of haemoglobin desaturation—did cause a reduced fatigue resistance (Morse et al. 2008) and exercise performance (Adir et al. 1999; Hirsch et al. 1985). The significance of the left-shift of the haemoglobin-dissociation curve for delivery of oxygen to the working mitochondria is further illustrated by the observation in dogs that the oxygen extraction was diminished by CO exposure in comparison to a similar reduction in oxygen-carrying capacity by 6% by exposure to hypoxic air that in fact enhanced the oxygen extraction (King et al. 1987). In line with the role of HbCO in the lower muscle fatigue resistance in smokers is the improvement of muscle fatigue resistance after two weeks of smoking cessation and a reversal of HbCO (Darabseh et al. 2021). Also the similar fatigue resistance in patients with COPD who had stopped smoking and age-, fat-free mass and physical activity-matched healthy controls (Degens et al. 2005) and enhanced maximal oxygen uptake by hyperoxic air if maximal exercise is associated with a reduction of Hb oxygen saturation (Nielsen 2003) are in line with the role of HbCO in muscle fatigue. It may well be that the presence of tissue regions with a PO2 < 7.5 mmHg (1 kPa)—not seen in muscles from non-smokers—that will reduce maximal mitochondrial oxygen consumption (Donnelly et al. 2022) contributes to the reduced fatigue resistance in smokers.

While our model shows that if the HbCO was increased to 6 or 20% the average PO_2_ was reduced by 1.9 and 7.2%, respectively, the tissue oxygenation was increased by 13.3% when a 29% reduction in maximal mitochondrial respiration (Ajime et al. 2021) was included in the model. Indeed, impaired mitochondrial respiration seen in peripheral blood mononuclear cells (Alonso et al. 2004) and peripheral lymphocytes (Cardellach et al. 2003) from smokers, muscle tissue in mice (Ajime et al. 2021) and lung epithelial cells exposed to cigarette extract (van der Toorn et al. 2007) will reduce oxygen extraction and increase tissue oxygenation, that will, combined with impaired oxygen delivery and detrimental effects of CO on the heart (Lippi et al. 2012) impair exercise capacity. However, the formation of HbCO (Darabseh et al. 2021) and the impaired mitochondrial function in smokers (Alonso et al. 2004; Cardellach et al. 2003) or in muscle cells exposed to smoke extract (Ajime et al. 2021) are readily reversible and may well underlie the improved muscle fatigue resistance after 2 weeks smoking cessation (Darabseh et al. 2021).

Our results of a similar tissue oxygenation profile between smokers (with smaller fibres, 6–20% HbCO, and a lower maximal mitochondrial respiration) and controls may also explain the absence of a rise in HIF1α protein in muscles from mice exposed to cigarette smoking (Ajime et al. 2021). Structural alterations, such as a slight muscle atrophy, might be interpreted as an adaptation to adequately avoid tissue hypoxia and maintain tissue oxygenation levels.

There are several limitations to this study. First, we assumed that blood flow in the working muscle was similar in smokers and non-smokers, even though smokers suffer from vascular dysfunction (Celermajer et al. 1993). The model assumed a homogeneous oxygen consumption throughout the tissue and an equal flow through each capillary. Flow heterogeneity has, however, a small impact on muscle oxygenation (Hoofd and Degens 2009), while the heterogeneity of capillary spacing, something that we did feed into the model, has a significant impact on tissue oxygenation (Degens et al. 2006a). Although maximal oxygen consumption of the muscle tissue was not measured directly, the SDH staining intensity has been shown to be strongly related to the maximal oxygen consumption of a fibre (van der Laarse et al. 1989). The 29% reduction in complex II respiration was seen in smoking mice, but this could not consider the impact of carbon monoxide on the respiratory chain and particularly complex IV. The reduction in maximal oxygen consumption may, thus, have been even more pronounced. However, the reduction is probably also related to the smoke exposure and in human muscle this effect may be less pronounced, but nevertheless in the same direction. Finally, the sample size is relatively small, but to obtain a significant different in tissue oxygenation with a statistical power of 0.80 between the non-smokers and smokers with 6% HbCO, we would need 30 people per group. This indicates that the morphological changes and the 6%HbCO in smokers have a minimal impact on tissue oxygenation. So, our model outcome may be quantitatively different from the real situation, but does give a qualitative indication of the impact of smoking on muscle oxygenation.

Overall, we conclude that while HbCO can explain the reduced exercise capacity after just one smoking session, an impaired mitochondrial respiration in chronic smokers is more important in reducing oxygen extraction and exercise capacity, with only a small contribution of the left-shift of the haemoglobin-dissociation curve. Both HbCO and impaired mitochondrial respiration are, however, fully reversible with smoking cessation.

## Data Availability

Data can be obtained from HD on request.

## References

[CR1] Adir Y, Merdler A, Ben Haim S, Front A, Harduf R, Bitterman H (1999). Effects of exposure to low concentrations of carbon monoxide on exercise performance and myocardial perfusion in young healthy men. Occup Environ Med.

[CR2] Ajime TT, Serre J, Wust RCI, Messa GAM, Poffe C, Swaminathan A, Maes K, Janssens W, Troosters T, Degens H, Gayan-Ramirez G (2021). Two weeks of smoking cessation reverse cigarette smoke-induced skeletal muscle atrophy and mitochondrial dysfunction in mice. Nicotine Tob Res.

[CR3] Alonso JR, Cardellach F, Lopez S, Casademont J, Miro O (2003). Carbon monoxide specifically inhibits cytochrome c oxidase of human mitochondrial respiratory chain. Pharmacol Toxicol.

[CR4] Alonso JR, Cardellach F, Casademont J, Miro O (2004). Reversible inhibition of mitochondrial complex IV activity in PBMC following acute smoking. Eur Respir J.

[CR5] Cardellach F, Alonso JR, Lopez S, Casademont J, Miro O (2003). Effect of smoking cessation on mitochondrial respiratory chain function. J Toxicol Clin Toxicol.

[CR6] Celermajer DS, Sorensen KE, Georgakopoulos D, Bull C, Thomas O, Robinson J, Deanfield JE (1993). Cigarette smoking is associated with dose-related and potentially reversible impairment of endothelium-dependent dilation in healthy young adults. Circulation.

[CR7] Darabseh MZ, Maden-Wilkinson TM, Welbourne G, Wust RCI, Ahmed N, Aushah H, Selfe J, Morse CI, Degens H (2021). Fourteen days of smoking cessation improves muscle fatigue resistance and reverses markers of systemic inflammation. Sci Rep.

[CR8] Degens H, Veerkamp JH (1994). Changes in oxidative capacity and fatigue resistance in skeletal muscle. Int J Biochem.

[CR9] Degens H, Sanchez Horneros JM, Heijdra YF, Dekhuijzen PNR, Hopman MTE (2005). Skeletal muscle contractile properties are unchanged in COPD patients with a normal fat-free mass. Acta Physiol Scand.

[CR10] Degens H, Deveci D, Botto-van Bemden A, Hoofd LJ, Egginton S (2006). Maintenance of heterogeneity of capillary spacing is essential for adequate oxygenation in the soleus muscle of the growing rat. Microcirculation.

[CR11] Degens H, Sanchez Horneros JM, Hopman MT (2006). Acute hypoxia limits endurance but does not affect muscle contractile properties. Muscle Nerve.

[CR12] Degens H, Gayan-Ramirez G, van Hees HW (2015). Smoking-induced skeletal muscle dysfunction: from evidence to mechanisms. Am J Respir Crit Care Med.

[CR13] Des Tombe AL, Van Beek-Harmsen BJ, Lee-De Groot MB, Van Der Laarse WJ (2002). Calibrated histochemistry applied to oxygen supply and demand in hypertrophied rat myocardium. Microsc Res Tech.

[CR14] Donnelly C, Schmitt S, Cecatto C, Cardoso LHD, Komlodi T, Place N, Kayser B, Gnaiger E (2022). The ABC of hypoxia—what is the norm. Bioenergetics Commun.

[CR15] Dorey A, Scheerlinck P, Nguyen H, Albertson T (2020). Acute and chronic carbon monoxide toxicity from tobacco smoking. Mil Med.

[CR16] Eisner MD, Anthonisen N, Coultas D, Kuenzli N, Perez-Padilla R, Postma D, Romieu I, Silverman EK, Balmes JR (2010). An official American Thoracic Society public policy statement: novel risk factors and the global burden of chronic obstructive pulmonary disease. Am J Respir Crit Care Med.

[CR17] Heishman SJ, Kleykamp BA, Singleton EG (2010). Meta-analysis of the acute effects of nicotine and smoking on human performance. Psychopharmacology.

[CR18] Hirsch GL, Sue DY, Wasserman K, Robinson TE, Hansen JE (1985). Immediate effects of cigarette smoking on cardiorespiratory responses to exercise. J Appl Physiol.

[CR19] Hlastala MP, McKenna HP, Franada RL, Detter JC (1976). Influence of carbon monoxide on hemoglobin-oxygen binding. J Appl Physiol.

[CR20] Hoofd L (1995). Calculation of oxygen pressures in tissue with anisotropic capillary orientation. I. Two-dimensional analytical solution for arbitrary capillary characteristics. Math Biosci.

[CR21] Hoofd L (1995). Calculation of oxygen pressures in tissue with anisotropic capillary orientation. II. Coupling of two-dimensional planes. Math Biosci.

[CR22] Hoofd L, Degens H (2009). The influence of flow redistribution on working rat muscle oxygenation. Adv Exp Med Biol.

[CR23] Kane LA, Ryan BJ, Schmidt W, Byrnes WC (2016). Acute, low-dose CO inhalation does not alter energy expenditure during submaximal exercise. Int J Sports Med.

[CR24] King CE, Dodd SL, Cain SM (1987). O_2_ delivery to contracting muscle during hypoxic or CO hypoxia. J Appl Physiol.

[CR25] Kizakevich PN, McCartney ML, Hazucha MJ, Sleet LH, Jochem WJ, Hackney AC, Bolick K (2000). Noninvasive ambulatory assessment of cardiac function in healthy men exposed to carbon monoxide during upper and lower body exercise. Eur J Appl Physiol.

[CR26] Larsson L, Orlander J (1984). Skeletal muscle morphology, metabolism and function in smokers and non-smokers. A study on smoking-discordant monozygous twins. Acta Physiol Scand.

[CR27] Lippi G, Rastelli G, Meschi T, Borghi L, Cervellin G (2012). Pathophysiology, clinics, diagnosis and treatment of heart involvement in carbon monoxide poisoning. Clin Biochem.

[CR28] McDonough P, Moffatt RJ (1999). Smoking-induced elevations in blood carboxyhaemoglobin levels. Effect on maximal oxygen uptake. Sports Med.

[CR29] Morris PB, Ference BA, Jahangir E, Feldman DN, Ryan JJ, Bahrami H, El-Chami MF, Bhakta S, Winchester DE, Al-Mallah MH, Sanchez Shields M, Deedwania P, Mehta LS, Phan BA, Benowitz NL (2015). Cardiovascular effects of exposure to cigarette smoke and electronic cigarettes: clinical perspectives from the prevention of cardiovascular disease section leadership council and early career councils of the American College of Cardiology. J Am Coll Cardiol.

[CR30] Morse CI, Pritchard LJ, Wust RC, Jones DA, Degens H (2008). Carbon monoxide inhalation reduces skeletal muscle fatigue resistance. Acta Physiol (Oxf).

[CR31] Mundel T, Jones DA (2006). Effect of transdermal nicotine administration on exercise endurance in men. Exp Physiol.

[CR32] Nielsen HB (2003). Arterial desaturation during exercise in man: implication for O2 uptake and work capacity. Scand J Med Sci Sports.

[CR33] Rietbrock N, Kunkel S, Worner W, Eyer P (1992). Oxygen-dissociation kinetics in the blood of smokers and non-smokers: interaction between oxygen and carbon monoxide at the hemoglobin molecule. Naunyn Schmiedebergs Arch Pharmacol.

[CR34] van der Laarse WJ, Diegenbach PC, Elzinga G (1989). Maximum rate of oxygen consumption and quantitative histochemistry of succinate dehydrogenase in single muscle fibres of *Xenopus laevis*. J Muscle Res Cell Motil.

[CR35] van der Laarse WJ, des Tombe AL, van Beek-Harmsen BJ, Lee-de Groot MB, Jaspers RT (2005) Krogh's diffusion coefficient for oxygen in isolated Xenopus skeletal muscle fibers and rat myocardial trabeculae at maximum rates of oxygen consumption. J Appl Physiol 99 (6):2173-2180 10.1152/japplphysiol.00470.2005 16051713

[CR36] van der Toorn M, Slebos DJ, de Bruin HG, Leuvenink HG, Bakker SJ, Gans RO, Koeter GH, van Oosterhout AJ, Kauffman HF (2007). Cigarette smoke-induced blockade of the mitochondrial respiratory chain switches lung epithelial cell apoptosis into necrosis. Am J Physiol Lung Cell Mol Physiol.

[CR37] Wittenberg JB (1970). Myoglobin-facilitated oxygen diffusion: role of myoglobin in oxygen entry into muscle. Physiol Rev.

[CR38] Wust RC, Jaspers RT, van der Laarse WJ, Degens H (2008). Skeletal muscle capillarization and oxidative metabolism in healthy smokers. Appl Physiol Nutr Metab.

[CR39] Wust RC, Morse CI, de Haan A, Rittweger J, Jones DA, Degens H (2008). Skeletal muscle properties and fatigue resistance in relation to smoking history. Eur J Appl Physiol.

[CR40] Wust RC, Jaspers RT, van Heijst AF, Hopman MT, Hoofd LJ, van der Laarse WJ, Degens H (2009). Region-specific adaptations in determinants of rat skeletal muscle oxygenation to chronic hypoxia. Am J Physiol Heart Circ Physiol.

