# Correction to: A modelling approach to disentangle the factors limiting muscle oxygenation in smokers

**DOI:** 10.1007/s00421-023-05314-0

**Published:** 2023-09-16

**Authors:** Hans Degens, Tomas Venckunas, Rob Cl Wüst

**Affiliations:** 1 Department of Life Sciences, Research Centre for Musculoskeletal Science and Sports Medicine, Manchester Metropolitan University, John Dalton Building, Chester Street, Manchester, M1 5GD UK; 2 Lithuanian Sports University, Kaunas, Lithuania; 3 Laboratory of Myology, Department of Human Movement Sciences, Faculty of Behavioural and Movement Sciences, Amsterdam Movement Sciences, Vrije Universiteit Amsterdam, Amsterdam, The Netherlands

**Correction to: European Journal of Applied Physiology (2023)** 10.1007/s00421-023-05289-y

The original version of this article unfortunately contained a mistake. Affiliation details for author Tomas Venckunas were incorrectly given as

Department of Life Sciences, Research Centre for Musculoskeletal Science and Sports Medicine, Manchester Metropolitan University, John Dalton Building, Chester Street, Manchester M1 5GD, UK

but should have been

Lithuanian Sports University, Kaunas, Lithuania

